# Mass Relapse Prevention to Reduce Transmission of *Plasmodium vivax*— A Systematic Review

**DOI:** 10.4269/ajtmh.22-0727

**Published:** 2023-12-20

**Authors:** Monica P. Shah, Nelli Westercamp, Kim A. Lindblade, Jimee Hwang

**Affiliations:** ^1^ Malaria Branch, Division of Parasitic Diseases and Malaria, Centers for Disease Control and Prevention, Atlanta, Georgia;; ^2^ Global Malaria Programme, World Health Organization, Geneva, Switzerland;; ^3^ U.S. President’s Malaria Initiative, Malaria Branch, Division of Parasitic Diseases and Malaria, Centers for Disease Control and Prevention, Atlanta, Georgia

## Abstract

Several temperate countries have used mass chemoprevention interventions with medicines of the 8-aminoquinoline class that prevent relapses from *Plasmodium vivax* before peak transmission to reduce transmission of malaria. The WHO commissioned a systematic review of the literature and evidence synthesis to inform development of recommendations regarding this intervention referred to as “mass relapse prevention” (MRP). Electronic databases were searched, 866 articles screened, and 25 assessed for eligibility after a full-text review. Two nonrandomized studies were included, one from the Democratic People’s Republic of Korea (391,357 participants) and the second from the Azerbaijan Soviet Socialist Republic (∼30,000 participants). The two studies administered a single round of primaquine over 14 days (0.25 mg/kg per day). From 1 to 3 months after the treatment round, the incidence of *P. vivax* infections was significantly lower in areas that received MRP than those that did not (pooled rate ratio [RR] 0.08, 95% CI 0.07–0.08). At 4 to 12 months after the treatment round, the prevalence of *P. vivax* infection was significantly lower in MRP villages than non-MRP villages (odds ratio 0.12, 95% CI 0.03–0.52). No severe adverse events were found. The certainty of evidence for all outcomes was very low and no conclusions as to the effectiveness or safety of MRP could be drawn. However, it is not likely that this intervention will be needed in the future as most temperate countries where *P. vivax* is transmitted are nearing or have already eliminated malaria.

## INTRODUCTION

*Plasmodium vivax* has the widest geographic range of any malaria parasite. Its life cycle includes a dormant liver stage (hypnozoite) that can cause relapses long after the initial infection.^1^ Strains of *P. vivax* from different geographic areas have been shown to have a range of relapse periodicities, with tropical strains relapsing more quickly and more often than temperate strains, perhaps because mosquitoes are present for only the summer months in temperate areas.^2^ Although treatment with antimalarial medicines from the 8-aminoquinoline class (hypnozoiticides, e.g., primaquine and tafenoquine) can effectively clear hypnozoites and prevent relapses, this class of antimalarials may increase the risk of hemolysis and acute hemolytic anemia (AHA) in people with glucose-6-phosphate dehydrogenase (G6PD) deficiency, who are more likely to reside in malaria-endemic areas.^3^^–^^5^ The increased risk of hemolysis and AHA is dependent on the degree of G6PD deficiency and on the dose and duration of exposure to an 8-aminoquinoline drug.

In some temperate settings where the degree and prevalence of G6PD deficiency is low and the relapse periodicity is long, chemoprevention with a hypnozoiticide immediately before the transmission season has been used to prevent relapses during periods of increased mosquito density and thus reduce *P. vivax* transmission. The first known use of an 8-aminoquinoline (quinocide, manufactured in the Union of Soviet Socialist Republics) in a mass treatment program occurred in Tajikistan between 1955 and 1956.^6^ The elimination of malaria in Tajikistan by the early 1960s was attributed in part to this program. Several other temperate countries administered primaquine to at-risk populations in an intervention known as “mass primaquine prophylactic treatment” (MPPT). The Democratic People’s Republic of Korea (DPRK) continued to use MPPT as part of its malaria elimination strategy as most recently as 2017.^7^^,^^8^ Although MPPT is a chemoprevention strategy that can be considered a form of mass drug administration (MDA), the intervention historically given pretransmission season did not include a medicine that clears blood-stage infection (schizonticide) and therefore is generally considered to be different from MDA, which provides a full therapeutic dose of an antimalarial medicine.

A WHO advisory group reviewed MDA and related interventions, including MPPT, in 2015.^9^ Although they concluded that MPPT was an effective intervention for *P. vivax*, the quality of the data was considered poor in many of the studies reviewed. The WHO Malaria Policy Advisory Committee ultimately recommended against use of MPPT for *P. vivax*, although whether this recommendation was related to safety concerns over lack of G6PD deficiency testing or other considerations was not made clear.

This review was undertaken at the request of the WHO to generate evidence for a guideline development group on malaria elimination interventions.^10^ Mass relapse prevention (MRP) was defined as administration of a full course of a hypnozoiticide to the entire population of a delimited geographic area to reduce *P. vivax* transmission. Given the recent U.S. Food and Drug Administration approval of a new 8-aminoquinoline medicine, tafenoquine, the intervention was renamed “mass relapse prevention” to remain agnostic as to the antimalarial medicine used.^11^

## MATERIALS AND METHODS

The methods for this systematic review have been described extensively elsewhere^12^ and in the protocol registered on PROSPERO (CRD42021240929, published April 4, 2021).^13^ Specific attributes of the methods for this review are noted here.

### Population, intervention, comparison, and outcomes.

The population included adults and children living in areas of ongoing or potential transmission of *P. vivax* including both tropical and temperate areas. Studies in special groups (i.e., refugees and soldiers) were included if they met eligibility criteria and constituted the entire population of a delimited geographic area. Mass relapse prevention was defined as the direct administration of a full therapeutic course (minimum of 3 mg/kg [22.5 mg × 8 days] administered within 8 weeks) of an antimalarial medicine that targets liver-stage parasites (i.e., an 8-aminoquinoline) at the same time to the entire eligible population of a defined geographic area, irrespective of the presence of symptoms or infection. Studies of chemoprevention in the form of individually timed intermittent preventive treatment in subpopulations (i.e., pregnant women, children, or infants), seasonal malaria chemoprevention (without an 8-aminoquinoline), and MRP targeted to a subset of the population based on age, occupation, or another demographic characteristic, and MDA with both a blood-stage schizonticide and a hypnozoiticidal drug were excluded from the review. The comparison was considered no intervention. Critical transmission-related outcomes were measured at the population level as previously described and included the incidence and prevalence of *P. vivax* infection.^10^ All adverse events as monitored and reported in the studies were abstracted including AHA and acute hemolysis. If outcomes were reported for unspecified *Plasmodium* species, the local epidemiology of the study area was used to infer the predominant species.

The pre-intervention or baseline period was defined as 1 to 12 months before, or time period concurrent with, the first round of MRP. For studies covering a single year, the post-intervention period refers to the time after the last or only round of MRP. For studies covering multiple years, each year was included separately, and the last round referred to the last round of each year. Post-intervention time periods were categorized, if available, as <1 month, 1–3 months, 4 to <12 months, and 12 to 24 months. If multiple data points were available within the same time period category and data could not be combined (i.e., nonindependent samples for prevalence or different cohorts for incidence), the latest measure within the time period was used.

### Contextual factors and operational parameters.

Evidence for contextual factors as defined in a previous paper was summarized if available.^12^ Insights from mathematical modeling on how variation in operational parameters alter the effectiveness of MRP were summarized for the following parameters if available: timing of rounds with respect to the transmission season; number of rounds; spacing of rounds; number of years for the intervention; coverage; adherence; and dosage and dosage schedule.

### Data collection and analysis.

**Figure 1.**
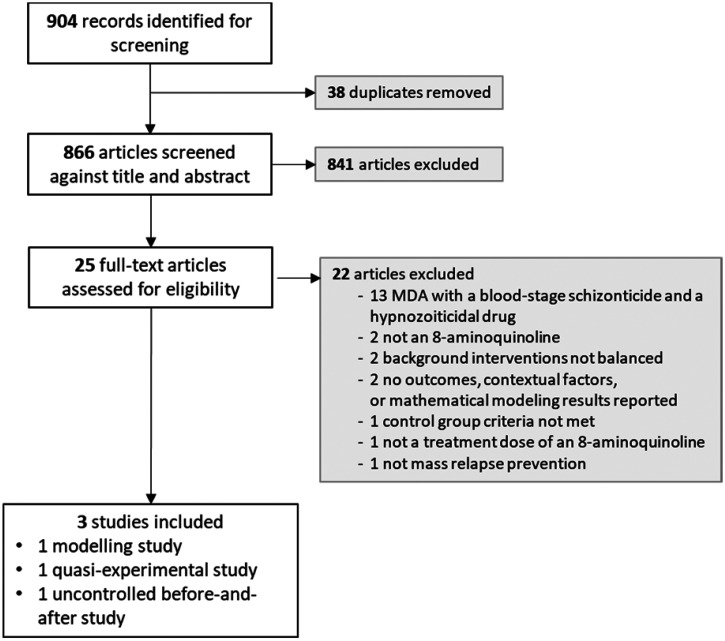
Preferred Reporting Items for Systematic Reviews and Meta-Analyses flow diagram. MDA = mass drug administration.

The selection of studies, data extraction, assessment of risk of bias, data synthesis, and the Grading of Recommendations Assessment Development and Evaluation (GRADE) process have been described previously.^12^ Given the extensive literature on MRP in Russian, all Russian-language articles were reviewed by a native Russian speaker (N. W.) to determine eligibility and to abstract data. Full-text studies that did not meet eligibility criteria are listed with their reasons for exclusion in Supplemental Table 2. A Preferred Reporting Items for Systematic Reviews and Meta-Analyses (PRISMA) flow diagram summarizing the study selection process is presented in Figure 1.

## RESULTS

A total of 904 articles was identified from searching electronic databases, registers, and other sources: 740 records from a database search from 2012 onwards (date of search: November 11, 2020), 154 from a previous review search before 2012,^14^^,^^15^ and an additional 10 from other sources. After de-duplication, 866 articles were screened for title and abstract eligibility, of which 25 were assessed for full-text eligibility criteria (Figure 1). A list of the excluded studies is available in Supplemental Table 2.

No cluster-randomized controlled trials and only two nonrandomized studies met criteria for inclusion: one quasi-experimental cluster-controlled study from the DPRK and one uncontrolled before-and-after study from the Azerbaijan Soviet Socialist Republic (ASSR).^16^^,^^17^ The characteristics of included studies are presented in Table 1. Both studies were conducted before 2003, and both were conducted in temperate areas where *P. vivax* has been reported to have a long relapse period and transmission is highly seasonal.^2^ The two studies administered a single round of primaquine over 14 days. One study reported a dosage of 0.25 mg/kg per day^16^; the other study did not report the dosage used, but it was inferred to be 0.25 mg/kg per day from a later report.^6^^,^^17^ In both studies, the single round of MRP was administered just before the peak malaria transmission season. The population targeted for MRP in the DPRK was 391,357 from 91 *ris* (villages); 85% coverage was achieved, and treatment adherence was 98%.^16^ Approximately 30,000 people were targeted for MRP in ASSR, but details on coverage and adherence were not provided.^17^

**Table 1:** Study characteristics

Reference	Country (years of intervention)	Baseline Transmission	Background Interventions	Dosage of Primaquine	G6PD Testing	Coverage (adherence)	Outcomes
Pant 2014^16^	Democratic People’s Republic of Korea, 2002–2003	*P. vivax* prevalence <1%	Not described	A single round of primaquine was administered over a 2-week period just before the peak transmission season; the dosage of primaquine was 0.25 mg/kg per day for 14 days	No testing was conducted, but historic data suggest G6PD prevalence was between 0.5% and 2.9%	85.3% (98.4%)	- Incidence—reported monthly from 1 to 5 months post-MRP - Prevalence—reported at <1 month and 4 months post-MRP - Adverse events reported
Rybalka 1979^17^	Azerbaijan Soviet Socialist Republic, 1970–1971	Not described	Not described	A single round of primaquine was administered over a 3-month period just before the peak transmission season; the dosage of primaquine was presumed to be 0.25 mg/kg per day for 14 days	Not described	Not described	- Incidence—reported monthly from <1 to 10 months post-MRP

G6PD = glucose-6-phosphate dehydrogenase; MRP = mass relapse prevention.

Both studies used standard 14-day dose regimens of primaquine, and neither conducted G6PD testing before administration of primaquine, although background rates of G6PD deficiency were considered low. These findings are therefore limited to temperate areas of *P. vivax* transmission where the strain exhibits a long relapse periodicity and G6PD deficiency prevalence is low.

**Figure 2.**
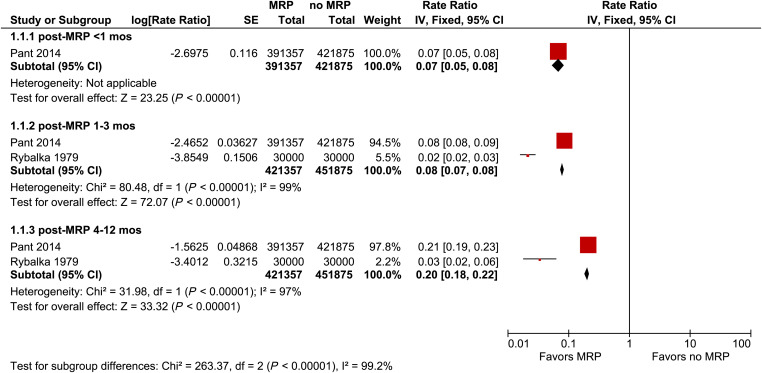
Forest plot of comparison: mass relapse prevention (MRP) versus no MRP on incidence of *Plasmodium vivax* infection.

Within 1 month of the start of the MRP program, malaria incidence was significantly lower in MRP villages compared with non-MRP villages in the DPRK (rate ratio [RR]: 0.07, 95% CI: 0.05–0.08, Figure 2). From 1 to 3 months post-MRP, the pooled RR for the incidence of malaria was 0.08 (95% CI: 0.07–0.08, *I*^2^ = 99%) suggesting a substantial reduction in malaria transmission. From 4 to 12 months post-MRP, the pooled RR was 0.20 (95% CI: 0.18–0.22, *I*^2^ = 97%), indicating reductions in incidence were sustained.

**Figure 3.**
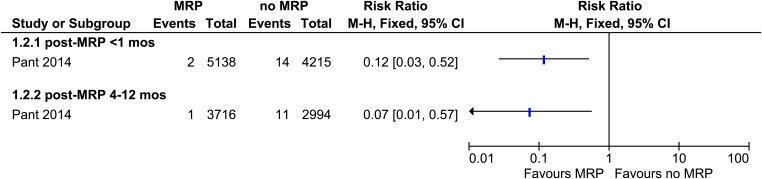
Forest plot of comparison: mass relapse prevention (MRP) versus no MRP on prevalence of *Plasmodium vivax* infection.

Only the DPRK study reported the prevalence of *P. vivax* infection.^16^ The prevalence of *P. vivax* immediately after MRP (<1 month) (odds ratio [OR]: 0.12, 95% CI: 0.03–0.52, Figure 3) and at 4 to 12 months post-MRP (OR: 0.07, 95% CI: 0.01–0.57) was significantly lower in MRP villages than in non-MRP villages.

The DPRK study provided data on adverse events, which were monitored only in the MRP villages by self-report using cards that were preprinted with possible side effects.^16^ No case of severe hemolysis or AHA was reported, and any side effect was reported by fewer than 4% of 400,000 cards completed. The most commonly reported side effects were headache (1%), epigastric pain (0.6%), nausea and vomiting (0.5%), dizziness (0.4%), and a change in urine color or black-colored urine (0.07%).

Information on contextual factors was provided by the DPRK study but limited to aspects of feasibility of implementing the intervention.^16^ The study in DPRK reported that the number of staff required to implement MRP for a population of 500 to 600 people was between seven and 14, and the team included doctors, senior health workers, and health volunteers.^6^

Both studies were nonrandomized and the ASSR study did not have a control group. Both studies lacked detail on potential confounding factors as well as information on recruitment, refusal rates, compliance, quality of diagnosis, or healthcare-seeking rates in the population (Supplemental Figure 1). As a result, the risk of bias for each study was considered high. The certainty of evidence for each outcome was assessed by the GRADE process as very low (Table 2), largely due to the high risk of bias in the studies.

**Table 2:** Grading of Recommendations Assessment Development and Evaluation summary of findings

Outcomes	Studies and Participants	Rate Ratio (95% CI)	Anticipated absolute effects* (95% CI)		Certainty
			Risk with no MRP	Risk with MRP	
Incidence < 1 month post-MRP	1 nonrandomized study (*n =* 813,232)	RR 0.07 (0.05–0.08)	36 per 1,000 person-years	3 per 1,000 (2–3)	Very low^†^^‡^^§^^ǁ^
Incidence 1–3 months post-MRP	2 nonrandomized studies (*n =* 873,232)	RR 0.08 (0.07–0.08)	111 per 1,000 person-years	9 per 1,000 (8–9)	Very low^†^^§^^ǁ^^¶^
Incidence 4–12 months post-MRP	2 nonrandomized studies (*n =* 873,232)	RR 0.20 (0.18–0.22)	13 per 1,000 person-years	3 per 1,000 (2–3)	Very low^†^^§^^ǁ^^¶^
Prevalence <1 month post-MRP	1 nonrandomized study (*n =* 9,353)	RR 0.12 (0.03–0.52)	0 per 1,000	0 per 1,000 (0–2)	Very low^†^^§^^ǁ^^¶^
Prevalence 4–12 months post-MRP	1 nonrandomized study (*n =* 6,710)	RR 0.07 (0.01–0.57)	4 per 1,000	0 per 1,000 (0–2)	Very low^†^^‡^^§^^ǁ^
Adverse events	1 nonrandomized study (*n =* 333,946)	Not estimable	0 per 1,000	0 per 1,000 (0–0)	Very low^†^^‡^^#^**

MRP = mass relapse prevention; RR = rate ratio.

* The risk in the intervention group (and its 95% CI) is based on the assumed risk in the comparison group and the relative effect of the intervention (and its 95% CI).

^†^ Downgraded by 2 due to risk of bias. Many risk of bias domains judged as high risk or not enough information to determine. High risk of bias due to confounding in both studies included for this outcome.

^‡^ Not downgraded for inconsistency due to single study result.

^§^ Not downgraded for indirectness because evidence was judged to be sufficiently direct for the domains of population, intervention, comparison, direct comparison, and outcome.

^ǁ^ Not downgraded for imprecision because lower and upper confidence limits indicate the same direction of effect.

^¶^ Not downgraded for inconsistency. Both studies provided the same direction and a similar magnitude (qualitatively) of effect.

^#^ Downgraded by 2 due to indirectness. Side effects were not measured or reported in the control group, so evidence is only provided in the intervention population.

** Not downgraded for imprecision because this criterion is not applicable for this outcome (no effect measure presented).

## DISCUSSION

The systematic review identified two nonrandomized studies that met the inclusion criteria for MRP. One study demonstrated a large decrease in measures of malaria transmission in intervention compared with control communities, whereas the other showed a large decrease in the incidence of malaria after the intervention compared with the period before. However, the very low certainty of evidence arising from studies that did not randomize the intervention, include appropriate controls, or provide sufficient detail of their methods prevent concluding whether MRP reduces malaria transmission.

Only one study conducted in a setting known to have a very low prevalence of G6PD deficiency collected information on potential adverse events, and the pharmacovigilance system was based on self-report. Although no cases of severe hemolysis were reported and the prevalence of side effects was generally low, the quality of evidence^18^ was rated as very low, and no conclusions can be drawn as to whether MRP increases harm to participants.

The magnitude of reductions in malaria outcomes measured by the two studies included in this review are similar to those reported elsewhere. Narrative reports of MRP from Azerbaijan, Tajikistan, North Afghanistan, and DPRK, which were excluded from the systematic review because they did not meet inclusion criteria, reported reductions in *P. vivax* malaria cases or incidence between 40% and 72%.^6^ Additionally, these studies recorded a low frequency of severe adverse events despite a population prevalence of G6PD deficiency as high as 39% in some areas. A *P. vivax* compartmental transmission model incorporating mosquito and human components in a low-endemic region of India where the local *P. vivax* strain averages 7 months to relapse suggested that high coverage with MRP should reduce transmission of *P. vivax*.^19^ The model estimated the burden of *P. vivax* to be substantially reduced after an annual mass treatment with a hypnozoiticide that reached 90% of the population, with effect sizes ranging from a 50% decline after the first annual intervention to 95% at the end of 5 years. After the MRP was halted in the simulation, the incidence gradually recovered over the subsequent 10 years.

However, although this review could not conclude whether MRP safely reduces malaria transmission, additional research on MRP is likely not warranted. The premise of MRP is to reduce hypnozoites just before a seasonal increase in the vector population; however, there are not many countries where MRP would be appropriate in the future given how few temperate countries remain with *P. vivax* transmission: Azerbaijan reported their last indigenous case of malaria in 2012, and Europe has been malaria free since 2015.^20^ Although MRP is not currently implemented in DPRK due to interruption of supplies following COVID-19 pandemic-related border closures (R. Premaratne, personal communication), the country reported fewer than 2,000 malaria cases in 2020 and is one of the 25 E-2025 countries considered to be approaching elimination.^21^ Furthermore, of the 15 countries that have been certified by WHO as malaria-free since 2000,^22^ more than half achieved that feat without deploying MRP.^6^

Although there is likely to be little need in the future for chemoprevention strategies that attempt to reduce transmission of *P. vivax* by deploying primaquine alone, given the few countries with highly seasonal *P. vivax* transmission that remain, there is evidence that primaquine’s effect on preventing relapses may be potentiated by co-administration with a schizonticide.^23^ The systematic review of MDA to reduce transmission of *P. vivax* included in this supplement identified only one study that included full treatment courses of both a schizonticide and primaquine.^24^ However, the WHO conditionally recommends the use of MDA to reduce *P. vivax* transmission and suggests that programs should reflect carefully on how to safely and feasibly administer a hypnozoiticide in addition to a schizonticide. Future efforts to evaluate MDA for *P. vivax* will need to investigate how to optimize and operationalize administration of both medicines.

## Supplemental Materials

10.4269/ajtmh.22-0727 Supplemental Materials
