# Supplementary material for: Mass Relapse Prevention to Reduce Transmission of Plasmodium vivax— A Systematic Review
Source: Am J Trop Med Hyg. 2023 Dec 20;110(4 Suppl):38–43. doi: 10.4269/ajtmh.22-0727 (PMC10993785; doi:10.4269/ajtmh.22-0727)
Supplement: Supplemental Materials [file tpmd220727.SD1.pdf]

Supplemental Data

Mass Relapse Prevention to Reduce Transmission of *Plasmodium vivax* - A Systematic Review

Monica P. Shah, Nelli Westercamp, Kim A. Lindblade and Jimee Hwang

Supplemental Table 1. Search strategy

Search Name: Cochrane Central Register of Controlled Trials

ID	Search
#2	"antimalarial":ti,ab,kw (Word variations have been searched)
#3	malaria:ti,ab,kw (Word variations have been searched)
#4	MeSH descriptor: [Malaria] explode all trees
#5	MeSH descriptor: [Antimalarials] explode all trees
#6	#2 or #3 or #4 or #5
#7	"mass chemoprophylaxis" or "mass drug administration" or "mass administration"
#8	"mass screening and treatment"
#9	"mass treatment"

Database: Embase 1947-Present, updated daily

1	malaria/ or malaria control/
2	antimalarial agent/ or antimalarial*.mp.
3	(malaria or antimalarial*).ab. or (malaria or antimalarial*).ti.
4	1 or 2 or 3
5	("mass chemoprophylaxis" or "mass drug administration" or "mass administration").mp.
6	mass drug administration.mp.
7	mass treatment.mp.
8	"mass screening and treatment".mp.
9	5 or 6 or 7 or 8
10	4 and 9

Pubmed Search history

**Search**	**Query**
#1	malaria Field: Title/Abstract
#2	antimalarial*or anti-malarial* Field:Title/Abstract
#3	(malaria[MeSH Terms]) OR antimalarials[MeSH Terms]
#4	#1 or #2 or #3
#5	((mass chemoprophylaxis) OR mass drug administration) OR mass administration Field:Title/Abstract

#6	"mass screening and treatment" Field: Title/Abstract
#7	(MDA[Title/Abstract] OR MSAT[Title/Abstract] OR iMSaT[Title/Abstract])
#8	mass drug administration[MeSH Terms]
#9	#5 or #6 or #7 or #8
#10	#4 and #9

Database : **LILACS**

Search on:	**malaria or antimalarial$ [Words] and mass administration [Words]**

Supplemental Table 2. List of studies excluded after full review and primary reasons for exclusion

Study	Reason for exclusion
Aliev 2000 [1]	Inadequate comparator arm: Background interventions not balanced in both arms
Aliev 2001[2]	Inadequate comparator arm: Background interventions not balanced in both arms
Cáceres 2004a[3]	Wrong intervention: MDA with a blood-stage schizonticide and a hypnozoiticidal drug
Cáceres 2004b[4]	Wrong intervention: MDA with a blood-stage schizonticide and a hypnozoiticidal drug
Cáceres 2005[5]	Wrong intervention: MDA with a blood-stage schizonticide and a hypnozoiticidal drug
Cáceres 2008[6]	Wrong intervention: MDA with a blood-stage schizonticide and a hypnozoiticidal drug
Chagin 1979[7]	Insufficient outcomes: No outcomes, contextual factors, or mathematical modeling results reported
Henderson 1934[8]	Wrong intervention: Not an 8-aminoquinoline
Hsiang 2013[9]	Wrong intervention: MDA with a blood-stage schizonticide and a hypnozoiticidal drug
Khaing 2016[10]	Wrong intervention: MDA with a blood-stage schizonticide and a hypnozoiticidal drug
Landier 2018[11]	Wrong intervention: MDA with a blood-stage schizonticide and a hypnozoiticidal drug
Levenson 1943[12]	Wrong intervention: Not an 8-aminoquinoline
Lysenko 1960[13]	Wrong intervention: Not mass relapse prevention
Lysenko 1973a[14]	Wrong intervention: MDA with a blood-stage schizonticide and a hypnozoiticidal drug
Lysenko 1973b[15]	Inadequate comparator arm: Control group criteria not met
Marasinghe 2020[16]	Wrong intervention: MDA with a blood-stage schizonticide and a hypnozoiticidal drug
Polevoy 1975[17]	Wrong intervention: MDA with a blood-stage schizonticide and a hypnozoiticidal drug
Singh 1968[18]	Wrong intervention: MDA with a blood-stage schizonticide and a hypnozoiticidal drug
von Seidlein 2019a[19]	Wrong intervention: MDA with a blood-stage schizonticide and a hypnozoiticidal drug
von Seidlein 2019b[20]	Wrong intervention: MDA with a blood-stage schizonticide and a hypnozoiticidal drug
Wojnarski 2016[21]	Wrong intervention: Not a treatment dose of an 8-aminoquinoline
Zhang 2014[22]	Insufficient outcomes: No outcomes, contextual factors, or mathematical modeling results reported

Notes: MDA = mass drug administration

Supplemental Figure 1. Risk of bias for included studies

	Failure to develop and apply appropriate eligibility criteria (inclusion of control population)	Flawed measurement exposure	Flawed measurement of outcome: Incidence	Flawed measurement of outcome: Prevalence	Flawed measurement of outcome: Adverse events	Failure to adequately control confounding	Incomplete follow-up
Pant 2014	+	+	?	−	−	−	+
Rybalka 1979	−	−	−			−	?
